# Machine Perfusion for Abdominal Organ Preservation: A Systematic Review of Kidney and Liver Human Grafts

**DOI:** 10.3390/jcm8081221

**Published:** 2019-08-15

**Authors:** Maria Irene Bellini, Mikhail Nozdrin, Janice Yiu, Vassilios Papalois

**Affiliations:** 1 Renal Transplant Centre, Belfast City Hospital, Belfast BT97AB, UK; 2 School of Medicine, Imperial College London, London SW72AZ, UK; 3 School of Medicine, University College London, London WC1E 6BT, UK; 4 Renal and Transplant Directorate, Imperial College Healthcare NHS Trust, London W120HS, UK; 5 Department of Surgery and Cancer, Imperial College London, London SW72AZ, UK

**Keywords:** machine perfusion, organ preservation, temperature, hypothermic, normothermic, transplant

## Abstract

Introduction: To match the current organ demand with organ availability from the donor pool, there has been a shift towards acceptance of extended criteria donors (ECD), often associated with longer ischemic times. Novel dynamic preservation techniques as hypothermic or normothermic machine perfusion (MP) are increasingly adopted, particularly for organs from ECDs. In this study, we compared the viability and incidence of reperfusion injury in kidneys and livers preserved with MP versus Static Cold Storage (SCS). Methods: Systematic review and meta-analysis with a search performed between February and March 2019. MEDLINE, EMBASE and Transplant Library were searched via OvidSP. The Cochrane Library and The Cochrane Central Register of Controlled Trials (CENTRAL) were also searched. English language filter was applied. Results: the systematic search generated 10,585 studies, finally leading to a total of 30 papers for meta-analysis of kidneys and livers. Hypothermic MP (HMP) statistically significantly lowered the incidence of primary nonfunction (PMN, *p* = 0.003) and delayed graft function (DGF, *p* < 0.00001) in kidneys compared to SCS, but not its duration. No difference was also noted for serum creatinine or eGFR post-transplantation, but overall kidneys preserved with HMP had a significantly longer one-year graft survival (OR: 1.61 95% CI: 1.02 to 2.53, *p* = 0.04). Differently from kidneys where the graft survival was affected, there was no significant difference in primary non function (PNF) for livers stored using SCS for those preserved by HMP and NMP. Machine perfusion demonstrated superior outcomes in early allograft dysfunction and post transplantation AST levels compared to SCS, but however, only HMP was able to significantly decrease serum bilirubin and biliary stricture incidence compared to SCS. Conclusions: MP improves DGF and one-year graft survival in kidney transplantation; it appears to mitigate early allograft dysfunction in livers, but more studies are needed to prove its potential superiority in relation to PNF in livers.

## 1. Introduction

The increasing demand for allografts and growing waiting lists have led to the utilisation of organs from extended criteria donors (ECDs) or organs with prolonged ischemic times [1]. These organs are associated with higher rates of discard due to an anticipated increased risk of primary non function (PNF) or delayed graft function (DGF); therefore, novel dynamic preservation technologies are increasingly being adopted with the aim to allow organ utilisation in these circumstances.

Dynamic preservation is not a novel concept yet: ex situ organ perfusion was introduced in 1934 by Charles Lindbergh and Alexis Carrel, who developed the first machine perfusion (MP) to preserve animal organs, but the first application in a human kidney was performed by Belzer in 1967. Although the initial result was successful, the concept of dynamic preservation was not pursued forward at that time, with a progressive utilisation of static cold storage (SCS) mainly for logistic and economic reasons.

In the last thirty years instead, with the change in demographics of the donor population and the idea of tailoring the preservation method to the single graft, the debate as to what is the optimal organ treatment prior to transplantation, along with the possibility to ideally let the parenchymal cells continue their metabolic activity before implantation, has led to a re-investigation of the technique of dynamic preservation [2]. In this scenario, where the temperature setting seems to be a main determinant for the subsequent cell activity, and with no evidence for the gold standard temperature to store retrieved grafts before implantation, there are two main modalities as alternatives to SCS: hypothermic (0–4 °C) or normothermic (34–37 °C) machine perfusion.

The aim of this study is to provide evidence with a systematic review and metanalysis of the outcomes in terms of organ viability and incidence of reperfusion injury in hypothermic/normothermic MP in comparison to SCS in kidney and liver human grafts.

## 2. Methods

The following search algorithm was adopted (Table 1):

### 2.1. Inclusion Criteria

All published studies including: abstracts from conferences, primary research on new preservation strategies, clinical trials (randomised controlled trials, non-randomised trials), retrospective studies (single centre study, cohort study), and case-controlled studies on organ transplantation of kidney and liver comparing normothermic machine perfusion (NMP) and/or hypothermic machine perfusion (HMP) to CS. To be included, the study had to analyse and discuss the effects of preservation temperatures on ≥1 following post-transplant outcomes. For kidneys: PNF, incidence and duration of DGF, serum creatinine post-surgery, one year graft survival, acute rejection, and estimated glomerular filtration rate (eGFR). For livers: PNF, serum bilirubin post-surgery, biliary stricture incidence, 1–7 day post-surgery peak AST and early allograft dysfunction (EAD).

### 2.2. Primary Objectives

- Compare DGF in transplanted kidneys (defined as the need for dialysis within 7 days post-transplantation) and EAD (defined using Olthoff [3] criteria) in transplanted livers preserved by MP to SCS.
- Compare PNF in kidneys and livers preserved by machine perfusion and simple cold storage.
- Compare post-transplantation estimated glomerular filtration rate (eGFR) and serum creatinine levels in kidneys preserved via HMP and SCS.
- Compare post-transplantation bilirubin and AST levels in serum in livers preserved via MP and SCS.

### 2.3. Secondary Objectives

- Where sufficient data existed, to compare one-year graft survival of organs perfused by MP and SCS.
- Compare acute organ rejection of organs preserved via MP and SCS.
- Indirectly compare the effectiveness of preserving liver grafts with HMP and NMP through evaluating studies that compared HMP to SCS and NMP to SCS.

### 2.4. Data Extraction and Review

Studies identified by the search strategy were screened for meeting the inclusion criteria using the titles and abstracts. Short-listed studies were further checked by reading the whole paper to exclude any ineligible studies, on the basis of the primary and secondary objectives.

### 2.5. Risk of Bias Assessment

The two reviewers (MN and JY) assessed the risk of bias independently. Randomised controlled trials (RCTs) and retrospective studies in humans were assessed by the Jadad scale. Where there was a disagreement about a Jadad score, advice from a third party (MIB) was sought.

### 2.6. Data Analysis

Meta-analysis was performed in Revman 5.3 [4]. The effect estimate was calculated together with 95% CI, studies were weighted by sample size, and heterogeneity was assessed with an I^2^ test. When I^2^ > 50%, a random effects model was used to account for heterogeneity, otherwise a fixed effects model was used. The summary effect was determined using the *p*-value calculated from the Z test. Odds ratio (OR) was used to compare dichotomous data in organs perfused by HMP/NMP to SCS.

Standardised mean difference (SMD) was used to compare continuous data. For the papers that did not report mean and standard deviation, the method suggested by the Wan et al. 2014 paper [5] was used to approximate mean and standard deviation values using the median and either the interquartile range or range reported in those papers. Studies where this method was used are marked by * in the forest plots.

## 3. Results

**Figure 1:**
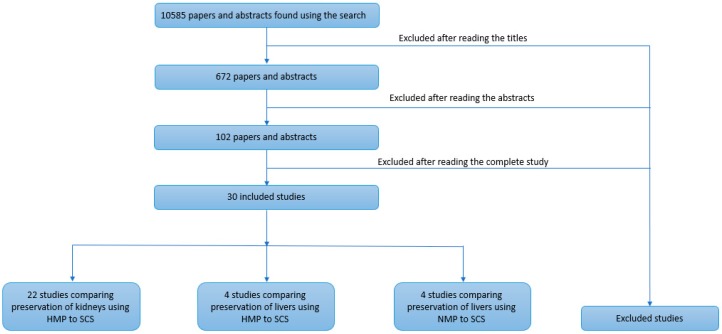
Flow diagram of the systematic literature search.

The systematic search generated 10,585 studies of which 672 abstracts and papers were shortlisted by reading the abstract title, and they were further reduced to 102 after reading the abstract. Finally, after reading the full article, a total of 30 papers were selected for meta-analysis (Figure 1).

### 3.1. Selected Study Characteristics

Twenty-two studies [6,7,8,9,10,11,12,13,14,15,16,17,18,19,20,21,22,23,24,25,26,27] identified by the systematic search were included into the analysis (Table 2); fifteen were published papers and seven were abstracts. Ten were RCTs [6,11,12,15,16,18,19,21,24,25], seven studies were retrospective [7,8,9,10,17,22,23], and five were prospective [13,14,20,26,27]. Predominantly, the studies used a LifePort^®^ kidney transporter for hypothermic machine preservation; there was a large variation in cold storage solution type, with some studies not mentioning the specific cold storage preservation solution, but instead referring to local guidelines.

The main difference between LifePort^®^ and RM3^®^ is that the latter provides oxygen by sweeping air over the membrane within the circuit.

Four studies identified in the systematic search were focused on comparing the effects of HMP and SCS in liver preservation [28,29,30,31] (Table 3). There was a lot of heterogeneity in the type of machine used for HMP of liver grafts; however, almost all studies had used the University Wisconsin solution for SCS.

Four normothermic perfusion of the liver vs SCS studies [32,33,34,35] were included in the meta-analysis (Table 4). The predominant machine perfusion device was OrganOx metra^®^. There were a variety of cold storage preservation solutions, and the most prevalent donor type was DBD (Table 4).

### 3.2. Risk of Bias Assessment

Overall studies had a poor Jadad score, and this is explained by many retrospective studies where organs preserved with MP were matched with organs preserved via SCS, so therefore no randomisation or blinding was possible. There was a significant proportion of RCT’s in the meta-analyses of HMP vs SCS in kidneys (Table 5) and NMP vs SCS in livers (Table 6); however, all of the studies comparing HMP to SCS in liver were retrospective studies and therefore had poor Jadad scales (Table 7).

### 3.3. Kidney Transplant Outcomes

PNF, DGF (incidence and duration), acute rejection, serum Creatinine, one-year graft survival, and e-GFR were meta-analysed.

### 3.4. Primary Non-Function

**Figure 2:**
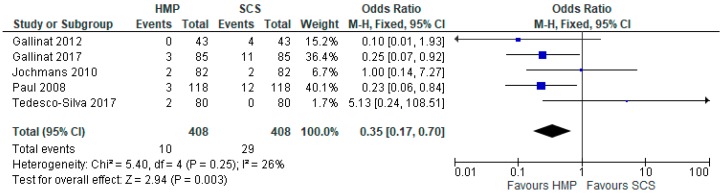
Primary nonfunction in kidneys preserved via HMP and SCS.

Five studies [12,13,15,21,24] which reported PNF (816 patients), demonstrated that HMP significantly decreased primary nonfunction compared to SCS (OR: 0.35 95% CI 1.02 to 2.53, *p* = 0.003) (Figure 2).

### 3.5. Delayed Graft Function

**Figure 3:**
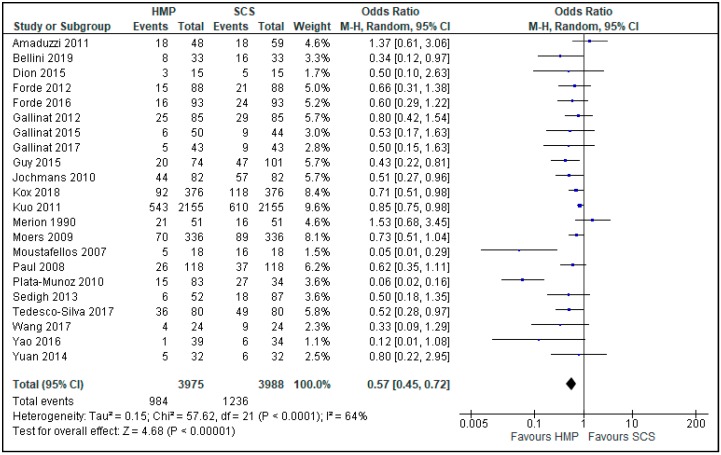
DGF in kidneys preserved by hypothermic machine perfusion and cold storage.

**Figure 4:**
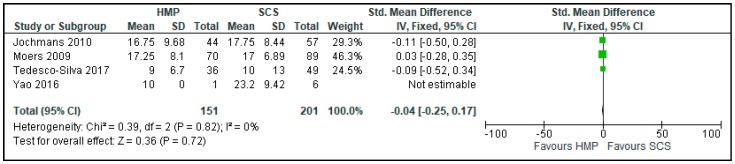
DGF duration in kidneys preserved by HMP and SCS. DGF duration was measured in days. In papers marked with “*”, mean and standard deviation were calculated using the method described by Wan 2014 [5].

Twenty-two studies [6,7,8,9,10,11,12,13,14,15,16,17,18,19,20,21,22,23,24,25,26,27] comparing HMP and SCS described the incidence of DGF (Figure 3), and its duration (Figure 4), with a total of 7963 patients. The overall OR was 0.57 (0.45, 0.72, 95% CI), with *p* < 0.00001, favouring a statistically significantly lower prevalence of DGF in kidneys preserved by HMP.

Four of the studies [15,19,24,26] reporting DGF were included in a meta-analysis comparing the duration of DGF (352 patients) (Figure 4). There was no difference in duration of DGF in kidneys preserved with HMP and SCS (SMD: −0.04 CI 95% −0.25 to 0.17, *p* = 0.72) (Figure 4).

### 3.6. Acute Rejection

**Figure 5:**
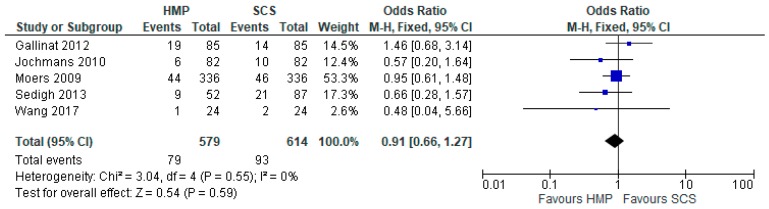
Acute rejection in Kidneys preserved via HMP and SC.

There was no significant difference in the prevalence of acute rejection in kidneys preserved by HMP or SCS (OR: 0.91 95% CI 0.66 to 1.27, *p* > 0.05). Five studies [12,15,19,23,25] were used for the meta-analysis of a total of 1193 patients (Figure 5).

### 3.7. Comparison of Serum Creatinine One Month after Kidney Transplantation

**Figure 6:**
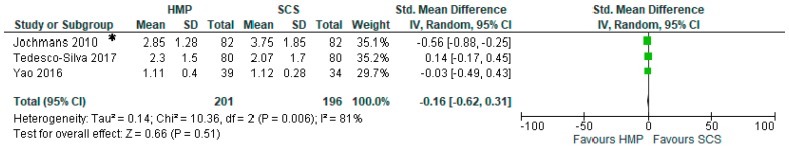
Comparison of one month post transplantation serum creatinine in kidneys preserved via HMP and SCS. In papers marked with “*”, mean and standard deviation were calculated using the method described by Wan 2014 [5].

Three studies [15,24,26] reported one-month post-transplantation serum creatinine (397 patients). There was no significant difference in serum creatinine values (SMD: −0.16 95% CI −0.62 to 0.31) (Figure 6).

### 3.8. One-Year Graft Survival

**Figure 7:**
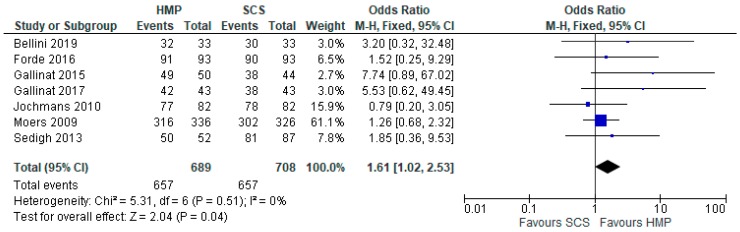
One year graft survival in kidneys preserved via HMP and SCS.

Seven studies [7,10,11,13,15,19,23] that had data on graft survival (1397 patients) were meta-analysed. Overall, kidneys preserved with HMP had a significantly longer one-year graft survival (OR: 1.61 95% CI: 1.02 to 2.53, *p* = 0.04) (Figure 7).

### 3.9. Post-Transplant Estimated Glomerular Filtration Rate in HMP and SCS Kidneys

**Figure 8:**
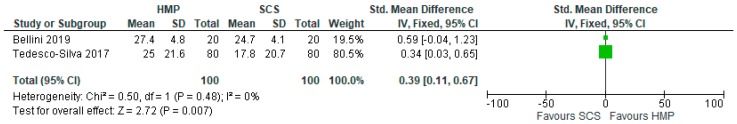
Estimated glomerular filtration rate (eGFR) in kidneys preserved via HMP and SCS; eGFR day 7.

**Figure 9:**
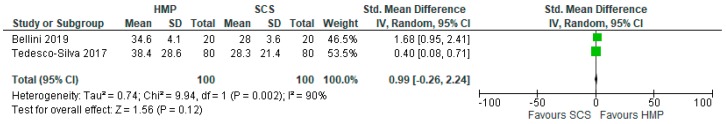
Estimated glomerular filtration rate (eGFR) in kidneys preserved via HMP and SCS; eGFR day 14.

**Figure 10:**
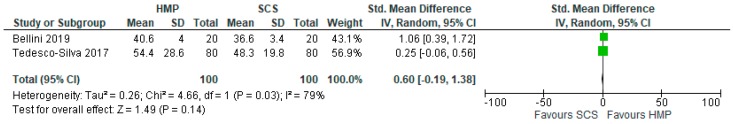
Estimated glomerular filtration rate (eGFR) in kidneys preserved via HMP and SCS; eGFR day 365.

One of our previous studies [7] as well as the one from Tedesco et al. [24] were the only two that reported eGFR at more than one time point after the surgery. Combined meta-analyses of 200 patients demonstrate that HMP increased eGFR only on day 7 post surgery (SMD: 0.39 95% CI 0.11 to 0.67, *p* = 0.007) (Figure 8). There was no significant difference in eGFR of kidneys preserved with HMP and SCS both on day 14 (SMD: 0.99 95% CI −0.26 to 2.24, *p* > 0.05) (Figure 9) and day 365 (SMD: 0.6 95% CI −0.19 to 1.38, *p* > 0.05) (Figure 10).

### 3.10. Liver Transplant Outcomes

PNF, EAD, and AST serum levels, bilirubin serum levels, and the incidence of biliary strictures were meta-analysed.

### 3.11. Primary Non Function

**Figure 11:**
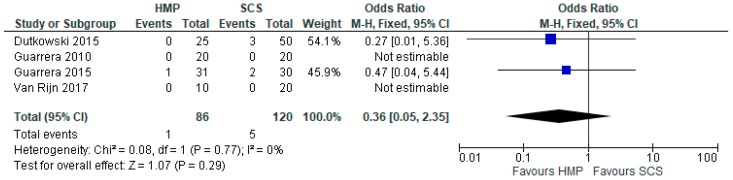
Primary nonfunction in livers preserved via HMP and SCS.

**Figure 12:**
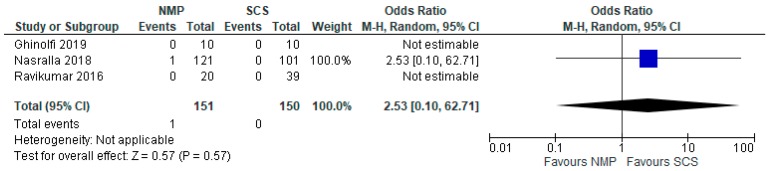
Primary nonfunction in livers preserved via NMP and SCS.

In livers preserved both by HMP (Figure 11) and NMP (Figure 12), there was no significant difference in PNF compared to livers stored using SCS. The odds ratio comparing HMP to SCS was 0.36 95% CI 0.05 to 2.35, *p* = 0.29, and the odds ratio comparing NMP to SCS was 2.53 95% CI 0.10 to 62.70, *p* = 0.67.

### 3.12. Early Allograft Dysfunction

**Figure 13:**
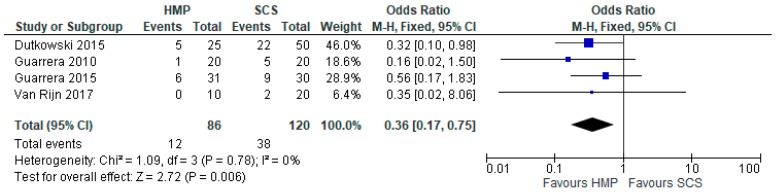
Early allograft dysfunction in livers preserved via HMP and SCS.

**Figure 14:**
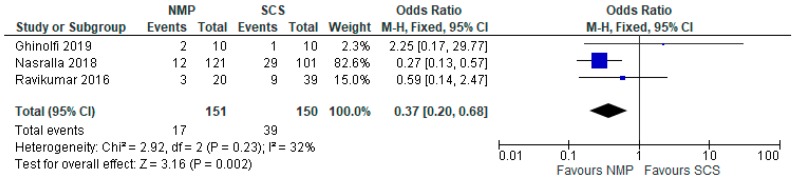
Early allograft dysfunction in livers preserved via NMP and SCS.

Four studies [28,29,30,31] compared EAD prevalence in livers stored using HMP and SCS (206 patients). Overall, livers stored with HMP showed lower prevalence of EAD (OR: 0.36 95% CI 0.17 to 0.75, *p* = 0.006) (Figure 13). Similar results were reported by the three studies comparing EAD prevalence in livers stored using NMP and SCS (301 patients). Overall, livers stored with NMP also showed lower prevalence of EAD compared to SCS (OR: 0.36 95% CI 0.17 to 0.75, *p* = 0.006) (Figure 14).

### 3.13. Serum AST

**Figure 15:**
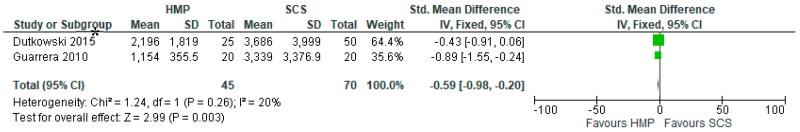
Peak serum AST in studies comparing HMP to SCS. In papers marked with “*”, mean and standard deviation were calculated using the method described by Wan 2014 [5].

**Figure 16:**
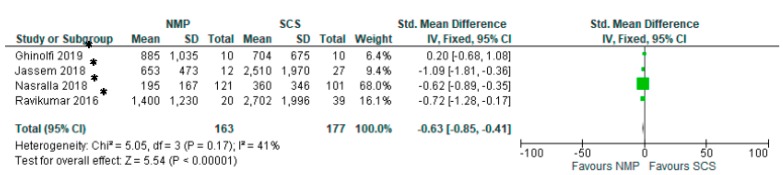
Peak serum AST in studies comparing NMP to SCS. In papers marked with “*”, mean and standard deviation were calculated using the method described by Wan 2014 [5].

Two studies [28,29] (115 patients) comparing HMP to SCS demonstrated the superiority of HMP in reducing post-transplantation AST levels (SMD −0.59 95% CI −0.98 to −0.20, *p* = 0.003) (Figure 15). Similarly, four studies [32,33,34,35] that focused on comparing NMP to SCS demonstrated that livers preserved with NMP had significantly lower serum AST levels than SCS (OR: −0.63 95% CI −0.85 to −0.41, *p* < 0.00001) (Figure 16).

### 3.14. Serum Bilirubin

**Figure 17:**
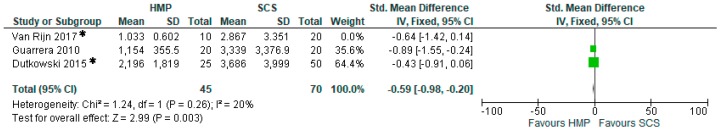
One week post transplantation peak serum total bilirubin in studies comparing HMP to SCS. In papers marked with “*”, mean and standard deviation were calculated using the method described by Wan 2014 [5].

Results from Dutkowski [28], Guarrera [29], and van Rijn [31] (115 patients) demonstrated the overall significant decrease in post transplantation serum bilirubin (SMD: −0.59 95% CI −0.98 to −0.20, *p* = 0.003) in livers stored with HMP compared to SCS (Figure 17).

**Figure 18:**
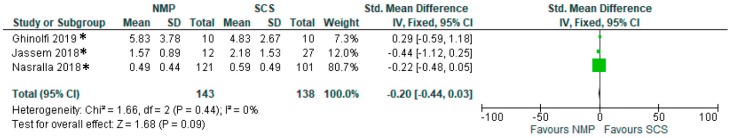
One week post transplantation peak serum total bilirubin in studies comparing NMP to SCS. In papers marked with “*”, mean and standard deviation were calculated using the method described by Wan 2014 [5].

Three studies [32,34,35] comparing NMP to SCS described total serum bilirubin one week post transplantation (181 patients), and demonstrated that there was no significant difference in bilirubin levels (SMD: −0.20 95% Ci −0.44 to 0.03, *p* = 0.09) (Figure 18).

### 3.15. Biliary Strictures

**Figure 19:**
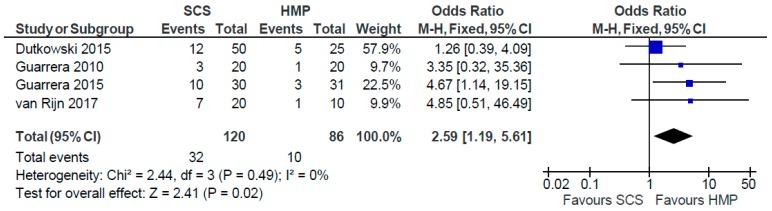
Post transplantation biliary stricture rates in studies comparing HMP to SCS.

Four studies [28,29,30,31] (Figure 19) comparing SCS to HMP in the preservation of livers (206 patients) demonstrated significant difference in incidence of post-transplantation strictures (OR: 2.59 95% CI 1.19 to 5.61, *p* = 0.02).

## 4. Discussion

This meta-analysis assessed the impact of dynamic preservation techniques on the viability and incidence of reperfusion injury in kidney and liver versus the traditional static cold storage before transplantation. The results were further sub-analysed in relation to the different organs considered.

HMP demonstrated significantly lowered delayed graft function incidence in transplanted kidneys compared to SCS, but it, however, had no effect on its duration, although only four studies reported this parameter. Furthermore, HMP was associated with reduced PNF and prolonged one-year graft survival, demonstrating the importance of machine perfusion technology in the utilisation of graft from extended criteria donors. Overall, serum creatinine of the transplanted grafts was similar, although a difference in eGFR could be seen on day 7 post transplantation. In the long term, there was yet no difference in kidneys preserved via HMP and SCS. This might lead to the debate of whether the long-term function of an organ is intrinsically related to the quality of the organ itself (standard or extended criteria), whilst the immediate post-transplant function is directly dependant on the preservation technique. For this reason, emergent possibilities of reconditioning during preservation are considered to improve the quality of the organ and to possibly impact the long-term outcome. In that regard, nutrients, therapeutic gases, mesenchymal stromal cells, gene therapies, and nanoparticles could be delivered to effectively repair an extended criteria organ during the preservation period and prior to implantation. The use of oxygen might in particular contribute to the long-term outcome of the preserved parenchymal cells. It is in fact of note, as shown in in Figure 10, that a difference in the one year eGFR is in favour of HMP kidneys preserved with an oxygenated circuit. Additional oxygen may support the aerobic activity and contrast the injury process of the cells with a more prominent effect in the long term. Furthermore, the efficiency of MP in assessing organ quality with possible reconditioning and predicting transplant outcome are of great interest in modern transplant practice, with an emerging role of these novel technologies to be evaluated as a possible diagnostic tool.

Differently from the kidney, no difference in PNF was seen in livers preserved via HMP or NMP compared to SCS; in liver preservation both HMP and NMP have demonstrated superior outcomes when it comes to mitigating early allograft dysfunction and post transplantation AST levels compared to SCS, but only HMP was able to significantly decrease serum bilirubin and the incidence of biliary strictures, compared to SCS. In addition to this, the value of AST as an end point is controversial because there can be a release of AST in the perfusate during MP; therefore, a more reliable marker should be considered in future studies. These conflicting results might be related to the relatively small numbers of RCT with, therefore, no sufficient evidence to conclude a clear superiority of one modality compared to the other. What appears to be clear is that more clinical studies are needed for verification with homogeneous parameters to measure the outcomes of interest.

In conclusion, there is growing evidence that MP allows for the utilisation of marginal kidneys with lower primary and delayed graft function rates. There is also evidence of improved early allograft dysfunction after dynamic preservation for livers, but more studies are needed to prove the potential superiority of these novel technologies.

## Figures and Tables

**Table 1:** Search Algorithm.

Step	Input
1	Machine perfusion and (Hypothermic or Normothermic)
2	(Organ* or kidney or liver) and (Preserv*)
3	1 and 2
4	Temperature and cell metabolism
5	3 or 4
6	Transplant*
7	exp Transplantation/
8	6 or 7
9	Renal* or kidney or liver or hepat*
10	(university of wisconsin or UW or HTK or histidine* or collins or hyperosmolar citrate or HOC or celsior or IGL-1 or institut-George* or custodial or belzer or MPS or KPS or marshall* or hypertonic citrate or soltran or ross)
11	8 and 9 and 10
12	5 or 11

**Table 2:** Studies comparing HMP and SCS in kidneys. Abbreviations: HTK: Histidine-tryptophan-ketoglutarate, UW: University Wisconsin, KPS-1: Kidney Perfusion Solution 1 (Organ recovery systems), SPS-1: Static Preservation Solution 1 (Organ recovery systems), ECD: expanded criteria donors, DBD: donation after brain death, and DCD: donation after cardiac death.

Study	Study Type	Machine	Cold Storage Preservation Solution	Donor Type	HMP Grafts (N)	Cold Storage Grafts (N)
Amaduzzi 2011(abstract) [6]	RCT	?	?	DCD	48	59
Bellini 2019 [7]	Retrospective	RM3^®^ Waters Medical System	?	DBD, DCD	33	33
Dion 2015 [8]	Retrospective	LifePort Kidney transporter^®^	?	DBD, DCD, ECD	15	15
Forde 2012 (abstract) [9]	Retrospective	LifePort Kidney transporter^®^	UW	DBD, ECD	88	88
Forde 2016 [10]	Retrospective	LifePort Kidney transporter^®^	UW	ECD	93	93
Gallinat 2012 [12]	RCT	LifePort Kidney transporter^®^	HTK or UW	DBD and DCD	85	85
Gallinat 2015 (abstract) [11]	RCT	?	?	ECD	50	44
Gallinat 2017 [13]	Prospective	LifePort Kidney transporter^®^	HTK or UW	DBD	43	43
Guy 2015 [14]	Prospective	LifePort Kidney transporter^®^	?	DCD, ECD	74	101
Jochmans 2010 [15]	RCT	LifePort Kidney transporter^®^	HTK or UW	DBD and DCD	82	82
Kox 2018 [16]	RCT	LifePort Kidney transporter^®^	HTK or CS-UW	DBD, DCD, ECD	376	376
Kuo 2011 (abstract) [17]	Retrospective	?	?	DCD, DBD	2155	2155
Merion 1990 [18]	RCT	MOX-100	Euro-Collins	DBD	51	51
Moers 2009 [19]	RCT	LifePort Kidney transporter^®^	HTK or UW or Euro-Collins	DBD and DCD	336	336
Moustafellos 2007 [20]	Prospective	LifePort Kidney transporter^®^	UW	DCD	18	18
Paul 2008 (abstract) [21]	RCT	?	?	ECD	118	118
Plata-Munoz 2010 (abstract) [22]	Retrospective	?	?	DCD	83	34
Sedigh 2013 [23]	Retrospective	LifePort Kidney transporter^®^	HTK, UW, Euro-Collins, Custodiol-N	ECD	52	87
Tedesco-Silva 2017 [24]	RCT	LifePort Kidney transporter^®^	SPS-1, Celsior preservation solution	DBD	80	80
Wang 2017 [25]	RCT	LifePort Kidney transporter^®^	?	DCD	24	24
Yao 2016 [26]	Prospective	LifePort Kidney transporter^®^	UW	DCD	39	34
Yuan 2014 (abstract) [27]	Prospective	LifePort Kidney transporter^®^	?	DCD	32	32

**Table 3:** Studies comparing HMP and SCS in liver.

Study	Study Type	Machine	Cold Storage Preservation Solution	Donor Type	HMP Grafts (N)	Cold Storage Grafts (N)
Dutkowski 2015 [28]	Observational	ECOPS device (Organ Assist)^®^	University Wisconsin	DCD, DBD	25	50
Guarrera 2010 [29]	Observational	Modified Medtronic PBS^®^	University Wisconsin	DCD, ECD	20	20
Guarrera 2015 [30]	Observational	Modified Medtronic PBS^®^	University Wisconsin	ECD	31	30
Van Rijn 2017 [31]	Observational	Liver Assist (Organ Assist) ^®^	According to local guidelines	DCD, DBD	10	20

**Table 4:** Studies comparing NMP and SCS in liver.

Study	Study Type	Machine	Cold Storage Preservation Solution	Donor Type	NMP Grafts (N)	Cold Storage Grafts (N)
Ghinolfi 2019 [35]	RCT	Liver Assist (Organ Assist)^®^	Celsior solution	DBD	10	10
Jassem 2018 [34]	Observational	OrganOx metra^®^	University Wisconsin	DBD	12	27
Nasralla 2018 [32]	RCT	OrganOx metra^®^	According to local guidelines	DBD, DCD	121	101
Ravikumar 2016 [33]	Observational	OrganOx metra^®^	University Wisconsin	DBD, DCD	20	39

**Table 5:** Risk of bias assessment of studies comparing HMP and SCS preservation in kidney.

Study	Randomisation	Randomisation Description	Inappropriate Randomisation	Double Blind	Double Blinding Description	Inappropriate Double Blinding	Description of Losses	Total Jadad Score
*Gallinat 2017*	0	0	0	0	0	0	1	1
*Forde 2016*	0	0	0	0	0	0	1	1
*Dion 2015*	1	0	−1	0	0	0	1	1
*Guy 2015*	0	0	0	0	0	0	1	1
*Gallinat 2012*	1	0	0	0	0	0	1	2
*Jochmans 2010*	1	1	0	0	0	0	1	3
*Merion 1990*	1	1	0	0	0	0	1	3
*Moers 2009*	1	1	0	0	0	0	1	3
*Moustafellos 2007*	0	0	0	0	0	0	1	1
*Sedigh 2013*	0	0	0	0	0	0	1	1
*Tedesco-Silva 2017*	1	1	0	0	0	0	1	3
*Bellini 2019*	0	0	0	0	0	0	1	1
*Wang 2017*	1	0	−1	0	0	0	1	1
*Yao 2016*	0	0	0	0	0	0	1	1
*Kox 2018*	1	0	0	0	0	0	1	2
*Gallinat 2015*	1	0	0	0	0	0	1	2
*Forde 2012*	0	0	0	0	0	0	1	1
*Amaduzzi 2011*	1	0	0	0	0	0	1	2
*Kuo 2011*	0	0	0	0	0	0	1	1
*Paul 2008*	1	0	0	1	1	0	1	4
*Plata-Munoz 2010*	0	0	0	0	0	0	1	1
*Yuan 2014*	0	0	0	0	0	0	1	1

**Table 6:** Risk of bias assessment of studies comparing HMP and SCS in liver.

Study	Randomisation	Randomisation Described	Inappropriate Randomisation	Double Blind	Double Blinding Description	Inappropriate Double Blinding	Description of Losses	Total Jadad Score
Dutkowski 2015	0	0	0	0	0	0	1	1
Guarrera 2010	0	0	0	0	0	0	1	1
Van Rijn 2017	0	0	0	0	0	0	1	1
Guarrera 2015	0	0	0	0	0	0	1	1

**Table 7:** Risk of bias assessment of studies comparing NMP and SCS in liver.

Study	Randomisation	Randomisation Described	Inappropriate Randomisation	Double Blind	Double Blinding Description	Inappropriate Double Blinding	Description of Losses	Total Jadad Score
Nasralla 2018	1	1	0	1	1	0	1	5
Ravikumar 2016	0	0	0	0	0	0	1	1
Jassem 2018	0	0	0	0	0	0	1	1
Ghinolfi 2019	1	1	0	0	0	0	1	3

## Abbreviations

AST	Aspartate transaminase
DGF	Delayed Graft Function
DBD	Donor after Brain Death
DCD	Donor after Cardiac Death
EAD	Early Allograft Dysfunction
eGFR	estimated Glomerular Filtration Rate
ECD	Expanded Criteria Donor
HMP	Hypothermic Machine Perfusion
HTK	Histidine-Tryptophan-Ketoglutarate
KPS-1	Kidney Perfusion Solution 1
MP	Machine Perfusion
OR	Odds Ratio
PNF	Primary Non-Function
RCT	Randomized Controlled Trial
SCS	Static Cold Storage
SMD	Standardised Mean Difference
SPS-1	Static Preservation Solution 1
